# Kuala Lumpur train collision during the COVID-19 pandemic

**DOI:** 10.1186/s13017-022-00405-3

**Published:** 2022-01-11

**Authors:** Alzamani M. Idrose, Fikri M. Abu-Zidan, Nurul Liana Roslan, Khairul Izwan M. Hashim, Saiyidi Mohd Azizi Mohd Adibi, Mahathar Abd. Wahab

**Affiliations:** 1 Department of Emergency Medicine, Hospital Kuala Lumpur, Kuala Lumpur, Malaysia; 2 Department of Surgery, College of Medicine and Health Sciences, UAE University, Al-Ain, UAE

**Keywords:** Mass casualty incident, Train, Collision, Disaster, Management, Triage, Communication, Malaysia

## Abstract

**Background:**

Two city trains collided in an underground tunnel on 24 May 2021 at the height of COVID-19 pandemic near the Petronas Towers, Kuala Lumpur, Malaysia, immediately after the evening rush hours. We aim to evaluate the management of this mass casualty incident highlighting the lessons learned to be used in preparedness for similar incidents that may occur in other major cities worldwide.

**Methods:**

Information regarding incident site and hospital management response were analysed. Data on demography, triaging, injuries and hospital management of patients were collected according to a designed protocol. Challenges, difficulties and their solutions were reported.

**Results:**

The train's emergency response team (ERT) has shut down train movements towards the incident site. Red zone (in the tunnel), yellow zone (the station platform) and green zone (outside the station entrance) were established. The fire and rescue team arrived and assisted the ERT in the red zone. Incident command system was established at the site. Medical base station was established at the yellow zone. Two hundred and fourteen passengers were in the trains. Sixty-four of them were injured. They had a median (range) ISS of 2 (1–43), and all were sent to Hospital Kuala Lumpur (HKL). Six (9.4%) patients were clinically triaged as red (critical), 19 (29.7%) as yellow (semi-critical) and 39 (60.9%) as green (non-critical). HKL's disaster plan was activated. All patients underwent temperature and epidemiology link assessment. Seven (10.9%) patients were admitted to the hospital (3 to the ICU, 3 to the ward and 1 to a private hospital as requested by the patient), while the rest 56 (87.5%) were discharged home. Six (9.4%) needed surgery. The COVID-19 tests were conducted on seven patients (10.9%) and were negative. There were no deaths.

**Conclusions:**

The mass casualty incident was handled properly because of a clear standard operating procedure, smooth coordination between multi-agencies and the hospitals, presence of a 'binary' system for 'COVID-risk' and 'non-COVID-risk' areas, and the modifications of the existing disaster plan. Preparedness for MCIs is essential during pandemics.

## Introduction

**Fig. 1:**
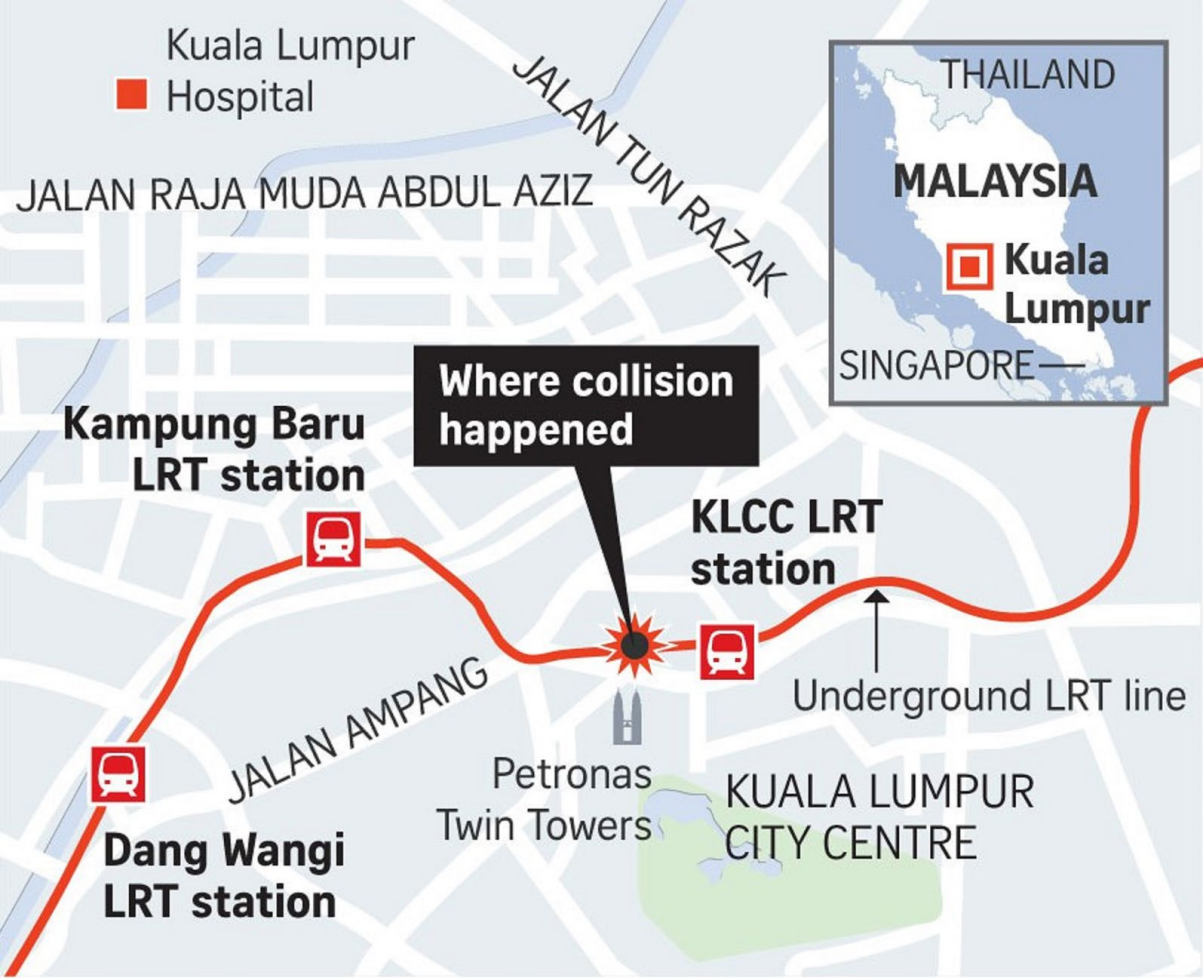
The incident site which occurred under the Petronas Twin Towers. It was 5 km from Hospital Kuala Lumpur which treated all 64 injured patients

The world is experiencing a large number of mass casualty incidents (MCI) which overwhelms the resources of health care systems and their capacity to respond to these incidents [1, 2]. The use of rail transport has increased globally with newer trains having higher speeds with better technology. An MCI of a train collisions may have high mortality because of the severe energy transfer to the victims of the incident [3]. The COVID-19 pandemic, which started in December 2019, has huge challenges for the medical response teams globally [4]. On 24 May 2021, two mass rapid transit trains collided in the underground station tunnel close to Kuala Lumpur City Centre (KLCC) Twin Towers, Malaysia, 25.7 m under the ground (Fig. 1). An empty train with one driver was travelling in the opposite direction of another train that was carrying 213 passengers. Both trains had a head-on collision.

**Fig. 2:**
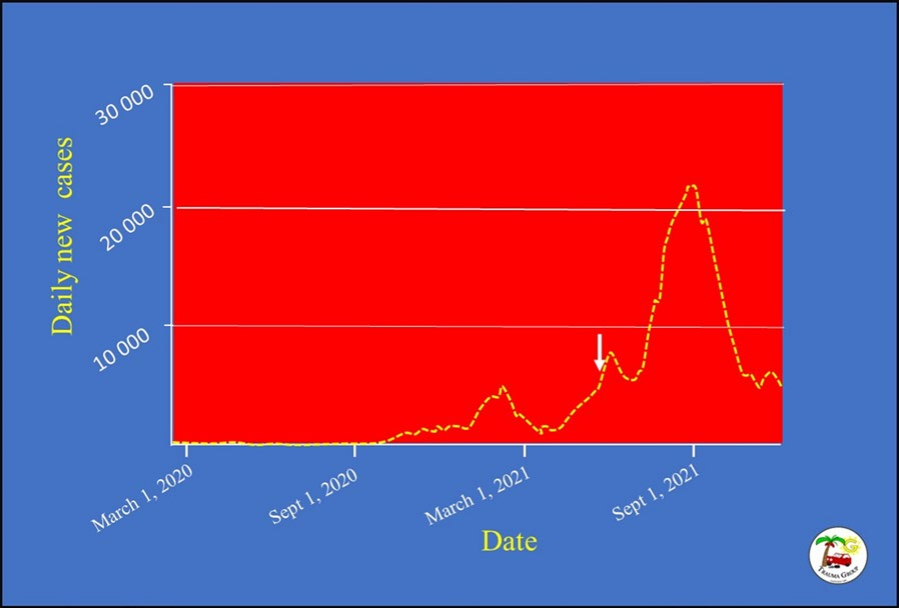
7-day moving average of new cases of COVID-19 in Malaysia during the pandemic. The train collision occurred on 24 May 2021 during the increasing trend of the second wave of the pandemic before its peak (white arrow). Data were retrieved from reference 6. Graph was drawn by Professor Fikri Abu-Zidan, College of Medicine and Health Sciences, UAE University, UAE

The incident occurred at night-time having a temperature of 35 °C. The location of the incident at a major city centre, the unanticipated event, the proximity to a crowded population and the COVID-19 pandemic with its high infection rate raised huge challenges for the management of this incident. The MCI occurred when the second wave of the pandemic was raising in Malaysia (Fig. 2). On that day, there were 6509 newly diagnosed COVID-19 cases, 6050 hospitalized patients and 921 patients admitted to the ICU. Out of 117 256 tests done at that day, 5.4% were positive. 4.9% of the population had at least one dose vaccine and only 2.08% were fully vaccinated [5, 6]. The hospitals in Kuala Lumpur were struggling to treat COVID-19 patients. We aim to evaluate the management of this mass casualty incident highlighting the lessons learned to be used in preparedness for similar incidents that may occur in other major cities worldwide.

## Methods

### Ethical consideration

The Medical Research and Ethics Committee, Ministry of Health, Malaysia, gave ethical approval for this study (Ref. number NMRR ID- 21-02187-UE0). Data were anonymous without any personal identifiers, were kept strictly confidential and were approached and analysed for the purpose of this study.

### Study and protocol design

This is a retrospective descriptive study. Data of all patients who were involved in the train mass casualty incident that happened in Kuala Lumpur (KL) on 24 May 2021 were collected according to a designed protocol which was developed following Professor Lennquist’s protocol [7]. This protocol aims to prospectively standardize the methods for reporting management of major incidents and disasters to be used for comparison, exchange of experiences and international collaboration. Furthermore, we have followed Howells et al.’s recommendations of mandatory major incident reporting within 6 months of their occurrence to document lessons learned and to facilitate future major incident planning [8].

### Subjects

All passengers and drivers (*n* = 214) who were involved in the train mass casualty incident that happened in KL on 24 May 2021.

### Data collection and studied variables

Studied data on the incident included triage, prehospital resources, hospital resources availability, hospital alert plan and response, coordination and command, communication system, total number and type of injuries, hospital load during response time, outcome and post-accident evaluation. Individual patient’s data included demography, vital signs, mechanism of injury, body injuries, radiological workup, surgical procedure, injury severity score (ISS), management and clinical outcome. The patients were followed up to 4 months after the incident.

### Statistical analysis

Data were presented as mean (SD) for continuous data, median (range) for ordinal data and number (%) for categorical data. Data were analysed using SPSS version 21.0 (IBM enterprise).

## Results

### Detailed description of the incident

A train broke down near the KLCC train station, and the automated driverless system was disabled. A driver was assigned to manually restart the empty train and move it away. The train engine could be started, and the driver moved it along the tunnel. There was a communication failure between the Operations Control Centre (OCC) and the driver. As a result, the train mistakenly entered the railway in the opposite direction of another incoming automated driverless train that was carrying 213 passengers. The driver saw the approaching train and reversed the train immediately. The incident occurred at 8.33 pm, in the tunnel that was 25.7 m under the ground. The location was 150 m from the Kula Lumpur City Centre (KLCC) train station. During the incident, the train that was carrying passengers was travelling at 40 kms per hour (km/h), whereas the manually driven train travelled at 20 km/h. Both trains collided head-on, causing passengers in the train, who did not have seat belts, to be thrown to the floor or hit other passengers, the steel poles, glass windows and walls of the train.

### Early response at incident site

Immediately after the incident, the first alarm reached the alarm centre of the operation centre at 8.33 pm. The incident site was very close to the KLCC train station. Internet signals were reachable, and phone calls could be made. The emergency call centre was alerted by both passengers and the train control centre. Live videos shared on the social media by the passengers indicated that the signals were functioning. When the incident occurred, there was blackout in the train. The emergency light came on subsequently. Agencies were notified within 5 min. Calls both from the railway operator and the public came to the 999 emergency call centre. Hospital Kuala Lumpur (HKL) received the call at 8.37 pm. The Fire and Rescue services were despatched along with their ambulances. Various other departments and agencies including the Police, Malaysian Civil Service and St John's Ambulance were alerted to respond. Private ambulances also voluntarily came despite not being alerted as photographs and videos of the incident were shared and became viral on the social media. The Malaysian National Security Council Directive 20 document mandates the Police as the overall On-Scene Commander. The Fire and Rescue Department becomes the Forward Field Commander. The leader of the team from the nearest Ministry of Health hospital becomes the ‘On-Scene Medical Commander’ (OMC). The OMC has the oversight and communicates with the Base Commander at Emergency Medical Call Centre (EMCC). OMC provides the inputs, risk assessment and communication regarding the incident. The decision to send to which hospital is made by the Base Commander after receiving the inputs from OMC.

**Fig. 3:**
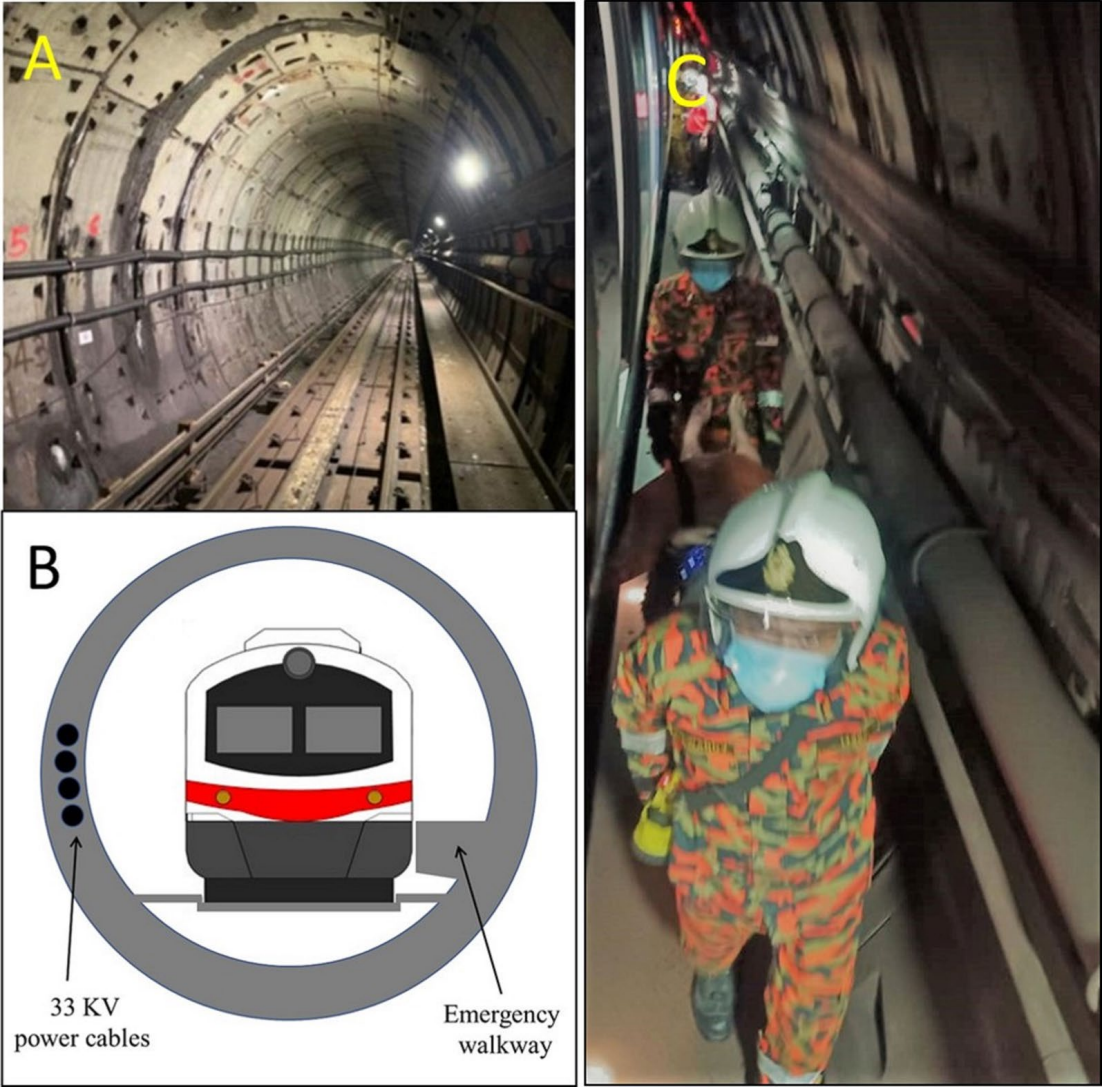
Schematic illustration of the train within the tunnel. The tunnel was 25.7 m under the ground. It was relatively dark, but there were light sources every 8 m (**A**). A narrow (0.6 m) maintenance walkway corridor was available on one side of the tunnel (**B**) that was used to carry the patients off the train (**C**)

The tunnel was relatively dark with light sources every 8 m (Fig. 3A). A narrow maintenance walkway corridor was available on one side of the tunnel (Fig. 3B, C). There were staircases and escalators leading to the ground floor passing an equivalent of 3-storey building with platform on every level. The tunnel ventilation machine was switched on automatically when the temperature reached 58 °C. Train movements at the area were stopped by the railway control centre. The railway's emergency response team was immediately alerted to respond before the arrival of other agencies. First, the team started to evacuate passengers who could walk out of the train. Those who could not walk were then evacuated by the personnel from Fire and Rescue Services. This was in line with the Simple Triage and Rapid Treatment (START) system which had been repeatedly practised in our annual exercise drills in the past. Prehospital personnel in Malaysia are trained and familiar with the START system.

#### Zoning at the incident site

**Fig. 4:**
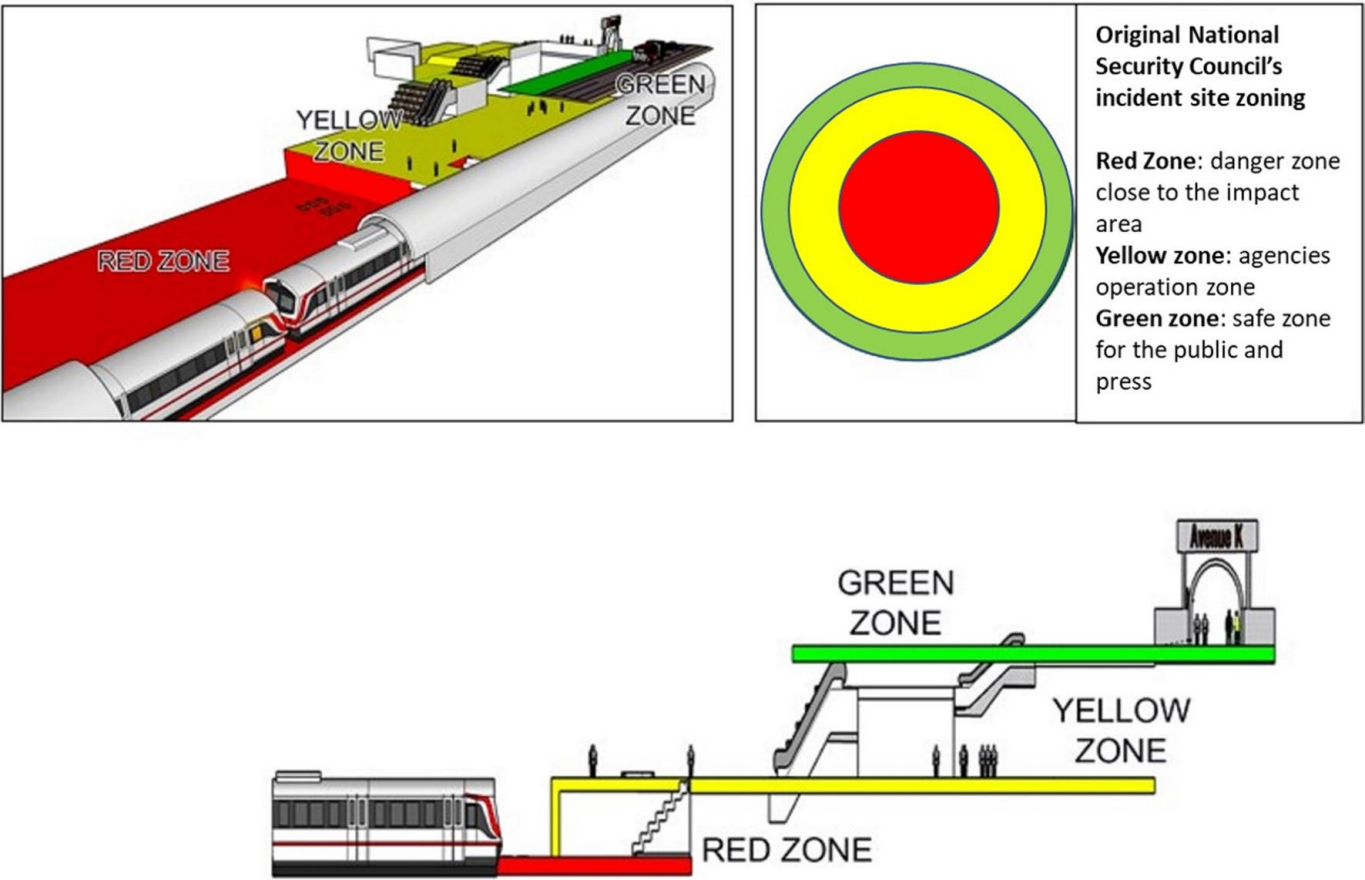
The zonings were established following the National Security Council's zonings recommendations. The classical circles (red, yellow and green zones) were modified. The red zone included the area from the train impact site, all the way to the first underground floor of the station platform. The yellow zone was established on the second underground floor and all the way to the station entrance. The green zone was established outside the station

**Fig. 5:**
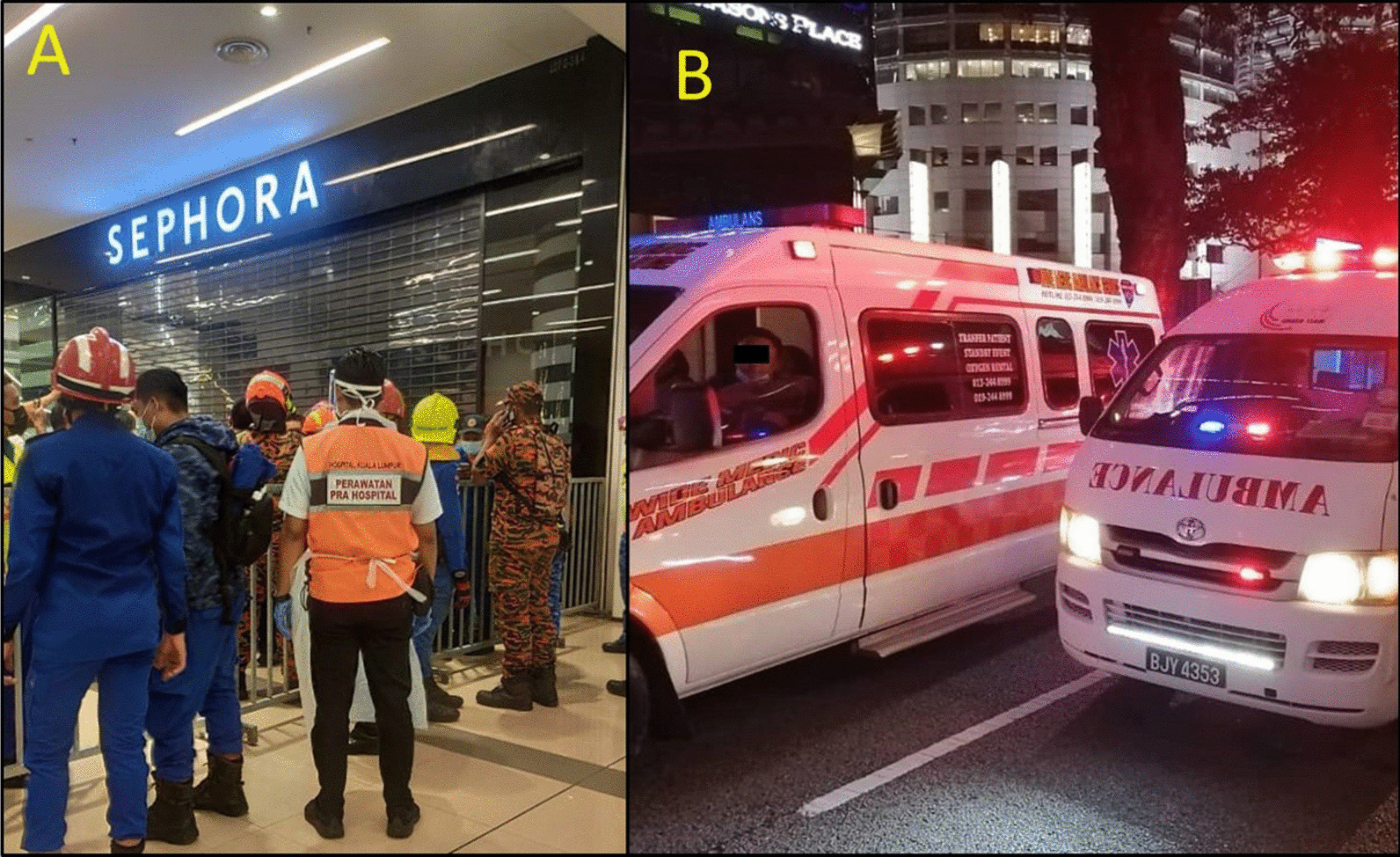
The border between the yellow zone and the green zone was controlled to permit only necessary personnel to enter the yellow zone (**A**). The 'green zone' was established outside the station where the transport station was located (**B**)

The zonings were established by the responders following the National Security Council's zonings recommendations (Fig. 4). Originally, the zonings were in circular shape with 'red zone' in the centre as the impact site, followed by the ‘yellow zone’ and ‘green zone’ furthest from the centre. These zones, respectively, represent dangerous, relatively safe and safe areas. Only those trained personnel equipped with protection equipment are allowed to enter the red zone. The yellow zone is for the establishment of the command post and the medical base station. The green zone is for the public and the press. This 'circular' pattern can be applied in flat surfaces of incidents. However, in this underground incident, modifications were made so that the 'red zone' was established from the train impact site, all the way to the first underground floor of the station platform. The 'yellow zone' was established on the second underground floor all the way to the station entrance. The 'green zone' was established outside the station (Fig. 5). Passengers had to walk on a very narrow platform (0.6 m wide) at the side of the tunnel to exit the tunnel. Rescue officers had to carry casualties who could not walk using stretcher on the same platform to the stairs up (Fig. 3C). The train crash site was only 150 m from the entrance to the KLCC train station, and therefore, patients could be carried to the station and brought up using the available wide staircases. Although elevators and lifts were functioning, they could not fit the stretchers.

#### Prehospital resources available and alerted

There were 14 ambulances on the ground (Table 1); one A ambulance, twelve B ambulances and two C ambulances. Type A ambulances are capable of providing advanced life support care and have equipment that can provide up to definitive airway intubation and ventilation. Type B ambulances are capable of providing basic life support, using supraglottic devices for airway management and other standard circulation interventions (like splints and intravenous fluid). They are manned by assistant medical officers or trained nurses helped by medical health attendants. Type C ambulances are manned by volunteer advanced first aiders from non-governmental organizations. They conduct minimal interventions and transport the patients to the most appropriate medical centres.

**Table 1:** Prehospital resources available/alerted

Unit	Number of teams	Types of Ambulances *	Distance (km) from the scene	Alerted (yes/no)	Alerted (time)		First unit on scene (time)
HKL prehospital medical team	1	A	5	Yes	8.37		8.55
Fire and Rescue Services	5	B	5	Yes	8.37		8.42
Malaysian Civil Defence	2	C	5	Yes	8.37		8.52
St John's Ambulance Malaysia	2	B	3	Yes	9.54		10.00
Private Ambulances	4	B	5	No	8.45		8.52

*Type of Ambulances: Type A: Advanced Cardiac Life Support; Type B: Ambulance equipped with Basic Life Support Equipment; Type C: Transport ambulances

Four governmental agencies despatched a median (range) of 2 (1–5) ambulances. There were four private ambulance teams that volunteered to attend to the scene without being alerted officially. All ambulances were at the scene within 10 min of the incidence. Ambulances usages were optimal and enough in transporting all non-ambulating critical and critical patients to the hospital. Some ambulating patients took private hailing cars to the hospital by their own choice.

### Response at hospital level

#### Hospital resources available and alerted

HKL is the primary responding hospital for any major incidents in Kuala Lumpur City Centre. It houses the Emergency Medical Call Centre (EMCC) which coordinates medical assistance. Hospital Ampang and Hospital Selayang are secondary responding hospitals in this region. HKL is the largest hospital in Malaysia and has a surge capacity that can handle 50 seriously injured MCI patients at any one time (Table 2). The initial information from the incident site reported that there were only 47 patients. Accordingly, a decision was made to send all injured patients to HKL. However, this was an underestimation as the final tally was 64 patients. Nevertheless, the hospital could handle this incident because majority of the patients were of 'green' category. In Malaysia, according to Emergency Medical and Trauma Services (EMTS) policy, triaging of cases is done based on colour code, whereby red is critical, yellow is semi-critical and green is non-critical. 'Stand down' was announced at 11.37 pm at the incident site (3 h and 4 min from the time of MCI).

**Table 2:** Hospital resources available/alerted

Name of hospital	Distance from scene	Total beds	ICU beds	Ventilators	Operating theatres	Trauma unit (yes/no)	Burn unit (yes/no)	Decontamination facility (yes/no)
Hospital Kuala Lumpur	5 km	2300	84	116	33	Yes	Yes	Yes
Ampang	5 km	562	10	52	5	Yes	No	Yes
Selayang	15 km	960	15	42	7	Yes	No	Yes

The Emergency Department managed the other acute patients simultaneously. The Department structure and capacity can manage an extra 50 patients even if they come at once or within short period of time without affecting the standard of routine acute care.

#### Coordination and command

HKL has a disaster plan for MCI management. As part of preparedness measures, simulations in the form of tabletop exercises as well as MCI drills were carried out every year. During the COVID-19 pandemic, however, drills could not be performed. Nevertheless, indoor simulations for disasters were held. The disaster plan is available to all personnel via the hospital's website. The plan activation involved the usage of the term 'red alert' which meant hospital staff response was required and 'yellow alert', for which staff were only required to be on standby mode to be called to the hospital if necessary. For 'red alert' there are two levels. Level 1 entails a response which mainly involves Emergency Department staff, additional key personnel from the hospital management, and the SONAR Departments (Surgery, Orthopedics, Anesthesiology, Neurosurgery and Radiology) personnel who should be available at the Emergency Department. Level 2, on the other hand, implies a response by staff from all departments in the hospital. During this incident, 'red alert Level 1' was activated. The hospital disaster plan incorporates elements of the incident command system. The Hospital Director is the 'Hospital Commander' who oversees the whole response operation. Under him are two coordinators: the clinical coordinator and the administrative coordinator. The clinical coordinator role is assumed by the head of Emergency Department and focuses on operations. The administrative coordinator heads the logistics, planning and financial matters. During the incident, the Emergency Zone Command Centre (EZCC) was the command post for all clinical activities in the Emergency Department which was headed by the emergency consultant on duty as 'EZCC Chief'.

#### Hospital alert plan and response

At HKL, the alert system was activated from the call centre to the emergency physician on duty. During the incident, the emergency physician updated the Head of Department (HOD) about the MCI situation. The alert was put into the department's management 'WhatsApp' group. The Hospital Director was informed about the incident by the HOD via a phone call. The Emergency Department and the hospital were immediately put under 'yellow alert' (on standby to receive patients). At the hospital, re-triaging was performed at the entrance based on the vital signs which were taken on arrival by triage officers. Then, the patient’s clinical acuity was made based on ABCDE (Airway, Breathing, Circulation, Disabilities and Exposure (limited)) resuscitation system. Furthermore, triage officers screened the patients for temperature and epidemiological link for COVID-19. The Emergency Physician of the triage zone decided which zone the patient should go to. Soon after that, the hospital received three critical patients followed by 12 other patients transferred by ambulances from multiple agencies. The level of alert was raised to 'red alert Level 1'. This was decided depending on the consultant's assessment and confidence that the department could handle the incident. The consultant assumed the role of EZCC Chief according to the disaster plan and took charge of the management of patients at the Emergency Department's treatment zones in the hospital.

Actions that were taken by the EZCC Chief were as follows: (1) briefing for all staff; (2) staff who were about to end their shift were asked to stay back to assist; (3) all patients from the incident had infectious disease triage including temperature check and epidemiological assessment. They were subsequently moved into 'dirty' or 'clean' areas based on the triage; (4) all patients from the MCI had black-coloured tags to differentiate them from other patients; (5) every Emergency Department zone (critical, semi-critical and non-critical) was extended while canvas beds were set up; (6) patients' particulars, diagnosis, progress and dispositions were regularly updated on a designated white board in every zone; (7) key specialties teams were alerted including Anaesthesiology, Neurosurgery, General Surgery and Orthopaedics; (8) hospital management team was alerted; (9) hospital bed managers were summoned, briefed and asked to prepare for bed disposition plans including ICU; (10) hospital operations room were alerted and prepared; and finally, a new area for X-rays imaging, in the building outside the Emergency Department was opened to cater for non-critical patients to speed up the imaging and avoid congestion which was a concern during the COVID-19 pandemic.

The Hospital Director and HOD joined the team within 30 min. The Hospital Director acted as the Hospital Commander, whereas the HOD assumed the role of Clinical Coordinator as per the disaster plan. The afternoon staff stayed back and joined the night team, doubling the number of staffs from all categories. There were also three volunteer emergency physicians who came to assist from home upon hearing about the MCI from social media. The Emergency Department personnel work in three shifts per day. Each staff is not allowed to work more than two continuous shifts because of the high intensity of the work. Once the patients are admitted to the ward, the care of the patients is continued by the ward staff. Most patients were discharged, and the number of patients admitted were within the capacity of the normal ward staff without the need to call for a backup from home.

All patients were handled smoothly, and the information of all patients was updated on a white board in each zone. The hospital bed managers managed to find ICU and ward beds for all patients requiring admissions. Two patients opted to go to private hospital for treatment. Key specialty teams from the Neurosurgery, General Surgery, Anaesthesiology and Orthopaedics came and planned managements for cases referred to them. The 'doubled' capacity of emergency staff could handle all the casualties with no further need to call for extra team from home. 'Stand down' in the Emergency Department was announced at 1.38 am, on 25 May 2021 (5 h and 1 min from the time of call received).

### HKL's binary system during the pandemic

Since the start of the pandemic, the Emergency Department in HKL operated with a binary system whereby the treatment zones were divided into 'clean' and 'dirty' areas. The details of this system have been recently published [9]. In short, with this system, the 'clean' areas catered for patients without symptoms of infectious disease, whereas the 'dirty' areas are for those who have them. Each of the 'clean' and 'dirty' areas has segments for red, yellow and green patients. This dual 'clean' and 'dirty' areas reflect the 'binary' term. For the 'dirty areas', there was also an isolation ward equipped with 14 negative pressure beds in the Emergency Department catering for patients who were COVID-19 positive [9].

**Fig. 6:**
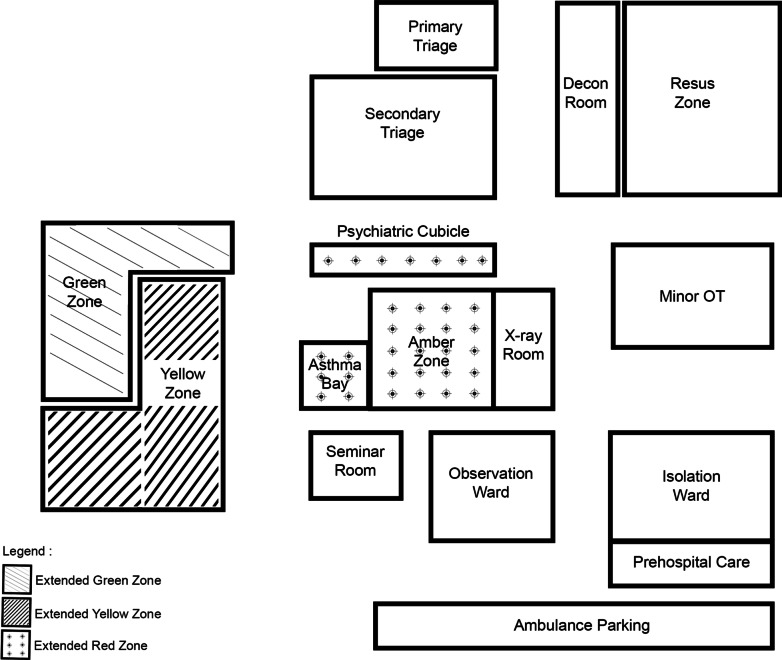
During the Mass Casualty Incident, the red, yellow and red zones of the 'clean' area in Hospital Kuala Lumpur were extended

During the MCI, the red, yellow and red zones of the 'clean' area were extended as shown in Fig. 6. None of the patients that came to the department had infectious disease symptoms nor epidemiological links. Therefore, all of them were treated in the extended 'clean area' that catered for the surge of patients. COVID-19 PCR tests were done on all seven admitted patients as part of the hospital protocol, while the discharged patients did not have the test done because they had no fever, no COVID-19 symptoms and no recent contact with a COVID-19 patient. Despite being in the 'clean' area, staff wore the minimum personal protective equipment (masks, face shields and gowns). On follow-up, none of the discharged treated patients had any COVID-19 symptoms.

#### Beds occupancy rate during the incident

At the time of the incident, HKL has an overall 52% bed occupancy rate. Bed capacity for patients was increased as an anticipation of increased COVID-19 cases. Elective surgery cases were stopped except for urgent and emergency surgery. ICU beds capacity was increased, and some of the operation theatres were converted into ICU beds.

### Clinical management of patients

There were 214 people in this incident, 213 passengers in the driverless train and 1 manual driver of the other train. Victims were evaluated by the medical staff at the incident site. Sixty-four (29.90%) were injured and sent to HKL. One hundred and fifty (70.09%) did not sustain significant injuries and were released home. The injured patients had a mean (SD) age of 32.9 (10) years; 33 (51.6%) were males. The median (range) ISS of those seen at the hospital was 2 (1–43); only five (7.81%) had an ISS of 16 and more. The median (range) ISS for the patients of red category was 22.5 (13–31); for the yellow category was 2 (1–6), whereas for the green category was 1 (1–2).

Table 3 shows the vital signs of the patients seen at the hospital. More than 60% were tachypnoeic (respiratory rate ≥ 20 per minute), less than 10% were tachycardic (heart rate > 120 beats per minute), and none was hypotensive. Two patients (3.1%) had severe traumatic brain injury (TBI), while one (1.6%) had moderate TBI. Soft tissue injuries were the most common type of injury which was sustained in 46 (48.4%) patients followed by chest injuries in 13 (13.8%) patients (Table 4).

**Table 3:** Vital signs at presentation of patients seen at  Hospital Kuala Lumpur who were injured during the Kuala Lumpur train collision on 24 May 2021 (*n* = 64)

Vital signs	Number	%
*Glasgow coma scale*		
13–15	61	95.3
19–12	1	1.6
< 9	2	3.1
*Respiratory rate*		
< 20	25	39.1
≥ 20	39	60.9
*Heart rate (bpm)*		
< 80	8	12.8
80–100	25	40
101–120	25	40
> 120	6	9.6
*Systolic blood pressure (mmHg)*		
90–120	25	40
121–139	22	35.2
140–159	13	20.8
160–179	3	4.8

**Table 4:** Injuries of the patients who were treated at Hospital Kuala Lumpur following the Kuala Lumpur train collision on 24 May 2021 (*n* = 64)

Injury	Number of patients	%
Soft tissue injury	46	48.4
Traumatic brain injury	6	6.3
Facial bone injury	2	2.1
Cervical or vertebra fracture	3	3.2
Chest injury	13	13.7
Intrabdominal injury	2	2.1
Extremity fracture	7	7.4
Dental injury	2	2.11
Perineum	1	1.05

Six (9.4%) patients were triaged as 'red' (critical), 19 (29.7%) as 'yellow' (semi-critical) and 39 (60.9%) as 'green' (non-critical). Seven (10.9%) patients were admitted to the hospital (3 to the ICU, 3 to the ward and 1 to a private hospital as requested by the patient). Fifty-six (87.5%) were discharged home. Only 6 (9.38%) had surgeries. This included craniectomy in two patients to evacuate an intracranial bleeding and elevation of a depressed skull fracture; one patient had thoracolumbar vertebral fracture fixations, one had open reduction and internal fixation (ORIF) for a Le forte fracture of the face; one had ORIF of a forearm fractures, one had intramedullary hip screw, and one had debridement of the soft tissue. The admitted patients stayed in the hospital for a median (range) 7 (1–28) days. None of them died. During the MCI response period, the psychologist team was not summoned because the MCI response was completed within 4 h from the time of arrival of the first patient. However, one patient was found to have depression and was referred for psychiatric consultation.

### The communication system

Malaysian government agencies which are involved in emergency management respond via a single '999' emergency number. The first call about the MCI was received from one of the passengers who was not injured. The Fire and Rescue Services Department was despatched within 5 min. Medical teams were on standby initially. Subsequently, when casualties were confirmed, ambulances from various agencies were despatched to the incident site. 'Government-integrated Radio Network' (GIRN) was used on the ground which works on Terrestrial Trunked Radio (TETRA) network. All governmental emergency response agencies are connected via this digital radio network. Walkie-talkies are also integrated via the same communication network among all relevant agencies. This included government hospitals, fire and rescue services, the police and civil protection agency. Hospitals use this system for communication with call centres of other hospitals and ambulance services. Internet-based messaging system in the form of 'WhatsApp' application was used internally in HKL to communicate with staff using 'group' created for department management in this MCI. It is a convenient method of communication. Nevertheless, if the Internet does not work, the call centre has a list of phone numbers to call key personnel for assistance or disseminating information. Table 5 describes the functionality of the communication systems utilized during the management of the current MCI. Walkie-talkie functioned well in almost all settings, while it was less optimal in the hospital. Telephones were not used in the prehospital setting.

**Table 5:** Functionality of the communication systems utilized during the management of the mass casualty incident

Unit	System and function score		
	Telephone	Walkie-talkie*	Internet-based texting
Ambulance	0	3	0
Prehospital teams	0	3	0
Hospitals	3	2	3
Fire and Rescue /Civil Protection	3	3	0
Police	3	3	0
Call centre	3	3	0

^*^Government integrated radio network

Score: 0 = not used, 1 = did not function, 2 = did function up to a point (unreliable), 3 = did function well

## Discussion

This study is unusual because it highlights the management of a disaster within another disaster. Both have different characteristics and management methods. Managing a head-on train collision that occurred deep in an underground tunnel during the COVID-19 pandemic had multiple challenges that have to be addressed. Even during a pandemic, hospitals should be prepared for MCIs [10]. The early information about this incident underestimated the actual number of victims. This influenced the decision by the command to send all patients to our hospital. Clarity of the event and correct timely information are important for proper decision-making [11]. Scarce information in the initial phase of MCIs is common. The information became clearer once the command post was set up at the current incident site. In addition, the integrated radio network of responders helped in centralizing all information to the call centre.

Factors affecting the decisions on distribution of victims in mass casualty incidents include the number of injured, the types of injuries, the available resources and the skills of the responders [12]. Almost 60% of severely injured patients of a train crash incident in Los Angeles in 2005 were transported to four community hospitals, while in another MCI that occurred in 2008, 93% of severely injured patients were transferred to a trauma centre [13]. During the pandemic, many elective operations in Neurosurgery, Orthopaedics and Plastic Surgery in HKL were halted, the surgical beds were transformed into COVID-19 wards, and the operation theatres were changed into intensive care units. HKL operated as a hybrid hospital, catering for both COVID and non-COVID patients. Surgical services were still running for emergency and semi-emergency cases. HKL was also the sole hospital running neurosurgical services in our region. This resulted in HKL receiving all the patients from the MCI. Patients will be directed to hospitals depending on available resources. The pandemic has forced the HKL hospital to practise a binary system that divided the Emergency Department into two identical areas: the dirty area for those having COVID-19 risk and the clean area for those having COVID-19 minimal risk. Each of these areas had red (critical), yellow (semi-critical) and green (non-critical) areas [9].

Reported train accidents that occurred during rush hours with almost similar number of passengers had more severe casualties. Major two train crash occurred in 1996, one in New York and the other in Washington. One occurred during the morning rush hours and resulted in 3 deaths and 162 injured victims, whereas the other occurred during the evening, out of rush hours, and resulted in 11 deaths and 114 injured patients [14]. The current MCI occurred in the late evening at the time of departure of people from work to their homes. Despite that, less death and number of serious injuries occurred. In the above MCIs that occurred in USA, two hospitals managed each incident compared with the current MCI in which only HKL managed it. The other two MCIs occurred at an open place, compared with the current MCI which was in a deep tunnel. The Reading train crash in the UK in 2006 carried 220 passengers, similar to the current MCI, 72 (33%) of them died. Sixty-one arrived to the hospital, only one of them died and 16 were admitted to the hospital. Of those, 10% had an ISS > 16 compared with 7.9% in the current MCI [6]. There were no deaths in the current MCI and relatively small number of severe injuries. In comparison with the Metrolink train crash in 2008 in Chatsworth, Los Angeles had 25 fatalities [15] who had severe chest and head injuries and were located in or near the locomotive.

Most of our patients (72%) in this incident had soft tissue injuries followed by chest injuries. This is similar to others [16]. We think that the mechanism of injury in the current train MCI is similar to those reported by Madsen et al. [17]. Majority of victims reported by Madsen et al. were thrown forward, thereby hitting parts of the loose and exposed seats in front [17]. Accordingly, the risk is higher when the passenger is facing forward in a moving train [17]. The train speed, the design of the concrete structure of the curve, the robustness of the carriage exterior, and the interior environment affect the injury severity [3]. In our situation, reducing the speed of one train which hit another train which was moving away from it decreased the energy transmission from the higher-speed train. This is proven by the fact that the carrier exterior was still intact**.** There was only a mechanical problem in the current MCI without fire or smoke. The train tunnels are generally small and narrow with closed air. The situation would have been much worse if there was fire or smoke in a closed or narrow place for the need of quick evacuation and ventilation.

Despite shortages in the communication, this MCI was handled with fairly fast response and coordination between multiple agencies at the incident and hospital levels. There was a clear command structure at the incident site. Similarly, the hospital has a clear disaster plan. Although a plan to accommodate MCI during the pandemic was not written, repeated discussions occurred among the Emergency Department managers in charge of disasters to agree on how to manage MCIs during the pandemic. The Emergency Medicine Department of HKL had multiple programmes of disaster medicine education in the past including international and national multi-agency annual drills. Drills and simulations improve the knowledge and skills of disaster response and are applicable in real-life situations [18]. Interestingly, a disaster drill in 2017 involved an incident scenario in an underground tunnel, similar to the current MCI. These exercises contributed to the relatively smooth and organized response to this incident.

One area of improvement in the management of this incident was in summoning staff for psychological first aid. Psychological support for both staff and patients is an integral part of our disaster management response, but it was not fully mobilized in this situation. This was partly due to the swiftness of handling the medical injuries. Exposure to such traumatic event could pose the risk of acute stress disorder and subsequent post-traumatic stress disorder (PTSD) if the symptoms persist beyond one month. Experiencing a train crash meant that the passengers had severe threat to life [19]. We did not explore any acute stress reactions among our staff. A study among rescue personnel following a train crash did not show acute stress reactions among them [20]. Nevertheless, this can be different in the time of the COVID-19 pandemic because the pandemic has major psychological impact on the population [21, 22]. A recent meta-analysis demonstrated that PTSD occurs in 33% in the population which was similar between the general population and the health care workers [23].

## Conclusions

This underground tunnel train collision at the height of the COVID-19 pandemic highlights the need to prepare for MCIs. Modification of disaster response plan is necessary in view of the current state of the pandemic. The 'binary' system that divided emergency department into 'dirty' and 'clean' areas in terms of COVID-19 risk enabled extension of areas to cater for MCI patients without affecting the routine COVID-19 management plan. All MCI patients during the pandemic who are sent to the hospital must be screened for the risk of COVID-19 and have a PCR test if the risk is high.

## Data Availability

There are no additional data available to share with the readers. Data can be shared with the Editor of the Journal if requested.

## Abbreviations

EMCC: Emergency Medical Call Centre
EMTS: Emergency Medical and Trauma Services
ERT: Emergency response team
EZCC: Emergency Zone Command Centre
GRIN: Government-integrated Radio Network
HKL: Hospital Kuala Lumpur
HOD: Head of Department
ISS: Injury Severity Score
KL: Kuala Lumpur
KLCC: Kuala Lumpur City Centre
MCI: Mass casualty incidents
OCC: Operations Control Centre
OMC: On-Scene Medical Commander
PTSD: Post-traumatic stress disorder
SD: Standard deviation
TBI: Traumatic brain injury
TETRA: Terrestrial Trunked Radio
